# Response: Commentary: *Borrelia miyamotoi*: 43 Cases Diagnosed in France by Real-Time PCR in Patients With Persistent Polymorphic Signs and Symptoms

**DOI:** 10.3389/fmed.2020.586694

**Published:** 2020-10-23

**Authors:** Michel Franck, Raouf Ghozzi, Julie Pajaud, Nadou E. Lawson-Hogban, Marie Mas, Alexis Lacout, Christian Perronne

**Affiliations:** ^1^ADNucleis, Grézieu la Varenne, France; ^2^Hôpital de Lannemezan, Service Infectiologie, Fédération Française contre les Maladies Vectorielles à Tiques, Lannemezan, France; ^3^Clinique Convert, Médecine Générale, Service des Urgences, Bourg en Bresse, France; ^4^Centre de diagnostic ELSAN, Centre Médico - Chirurgical, Aurillac, France; ^5^Hôpital Universitaire Raymond Poincaré (Assistance Publique – Hópitaux de Paris), Département d'Infectiologie, Université de Versailles – Saint Quentin, Paris-Saclay, Garches, France

**Keywords:** *Borrelia*, *Borrelia miyamotoi*, Lyme, post treatment Lyme disease syndrome, persistent polymorphic syndrome possibly due to a tick bite

We read with interest Wagemakers et al.'s reply criticizing our discovery of *Borrelia miyamotoi* in France (1). These commentaries are scientifically totally irrelevant for the following reasons:

From a clinical point of view:

Wagemakers et al. pretend that the population is not sufficiently characterized. Our patients were seen in consultation (outpatient in private practice), by doctors trained in the diagnosis of SPPT (Persistent polymorphic syndrome possibly due to a tick bite), and PCR tests were performed when they met the precise definition of SPPT. The SPPT officially recognized in France by the High Authority for Health (HAS) is, however, precisely defined by a clinical triad associating several times a week, for more than 6 months, with exclusion of other possible co-morbidities (neoplasia or some auto-immune disorders, for example): a polyalgic syndrome (musculoskeletal pain and/or neuropathic pain and/or headaches); persistent fatigue with reduced physical capacities; cognitive complaints; a possible history of tick bite (2). Our questionnaire included the items published in a reference cited in the article (3). We acknowledge that although other possible diagnoses have been formally excluded, these could have been collected more systematically. The difference between SPPT and PTLDS (Post Treatment Lyme Disease Syndrome) is that a diagnosis of Lyme disease has not been proven, principally because the efficiency of Lyme serology lacks sensitivity, which is established by several publications (4, 5). We agree that the problem is certainly more complex, as patients are often poly-infected and therefore borreliosis (including *B. miyamotoi*) probably represents the tip of an iceberg. SPPT patients (unlike PTLDS) also may have not been treated.

May we put forward that a history of a tick bite is not necessary for the diagnosis of SPPT, as in many cases the tick bite is unnoticed (e.g., small tick, bites in folds, in inaccessible areas of the body).

Last but not least, the clinical signs in the control group have been described, contrary to what is said in the comments: “*B. miyamotoi* was searched by qPCR on a control group of 24 healthy asymptomatic students” (Table 3)! For all these reasons, we cannot accept the clinical criticisms of Wagemakers et al.

From a biological point of view:

Wagemakers et al. discuss the administrative background of Franck et al. research. Such an argument should have no room in a scientific commentary but is also irrelevant. The study used detection kits made by an officially ISO certified laboratory: ISO 13485.

Wagemakers et al. speak of possible contaminations. This possibility is ruled out by the preparation of mixes in a DNA-free room, by the absence of any non-conformity event published by this laboratory, and by the systematic negative controls on each qPCR plate. Moreover, any contamination by a mastermix or by an amplicon liberation due to the qPCR plate opening is excluded.

Wagemakers et al. falsely claim that human European are variants of Asian strains and must necessarily have a thymidine at position 26, while 6 out of 7 patients in the Franck et al. study have a cytosine. Actually, several observations of a cytosine at position 26 have been described in Europe in Poland (GenBank MK674170), in Estonia (GenBank KX418610), and in Russia (GenBank MK955927 and GenBank KU169374) (Figure 1).

**Figure 1:**
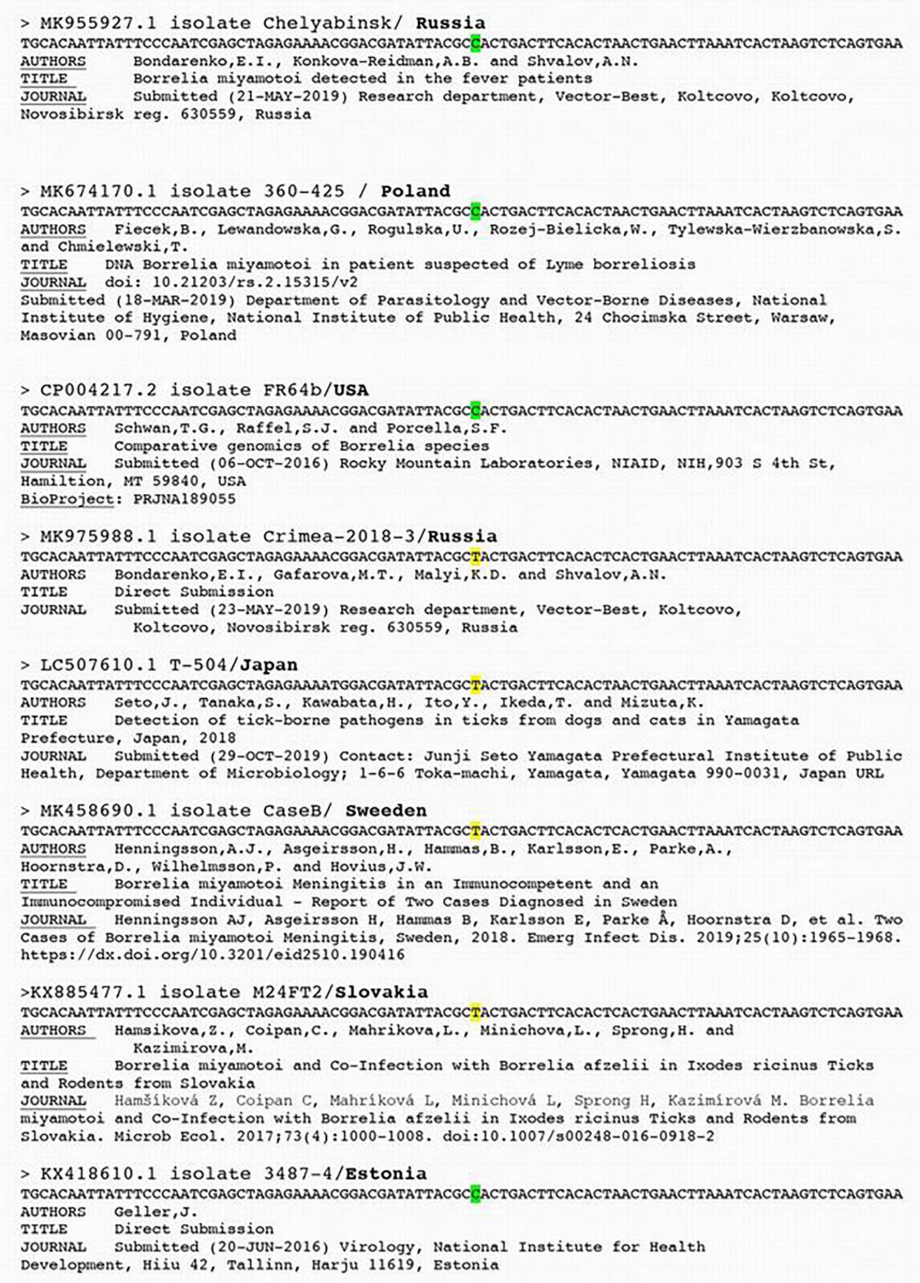
*Borrelia miyamotoi* isolates DNA sequences alignment (source Genbank).

The arguments of Wagemakers et al. leading them to reject our series and the discovery of *Borrelia miyamotoi* (1) have no scientific basis, (2) show a lack of familiarity with the clinical signs of patients presenting with tickborne disease, (3) suspect, without any well-founded argument, poor technical procedures.

## Author Contributions

All authors listed have made a substantial, direct and intellectual contribution to the work, and approved it for publication.

## Conflict of Interest

MF, JP, and NL-H were employed by the company ADNucleis. The remaining authors declare that the research was conducted in the absence of any commercial or financial relationships that could be construed as a potential conflict of interest.
